# Leveraging Healthcare Analytics to Uncover Social Determinants of Health Disparities in Congestive Heart Failure

**DOI:** 10.7759/cureus.98720

**Published:** 2025-12-08

**Authors:** Ifeoluwa R Solaru, Laura Ikuma, Tonya Jagneaux, Isabelina Nahmens

**Affiliations:** 1 Healthcare Engineering, Louisiana State University, Baton Rouge, USA; 2 Industrial Engineering, Louisiana State University, Baton Rouge, USA; 3 Medicine, Louisiana State University Health Sciences Center, Baton Rouge, USA

**Keywords:** congestive heart failure, healthcare analytics, machine learning, predictive analytics, social determinants of health

## Abstract

Background

Congestive heart failure (CHF) is shaped by both clinical risk factors and social determinants of health (SDOH), which influence disease progression, medication adherence, and care access. As healthcare systems incorporate SDOH screening into routine electronic health record (EHR) workflows, understanding the relationship between social needs and CHF outcomes is critical for risk stratification and population management.

Methods

A retrospective analysis was conducted using 30,534 inpatient EHR records, including 7,618 patients with CHF and 22,916 without CHF. Associations between CHF status and SDOH variables were examined using chi-square tests. Multivariable logistic regression evaluated adjusted predictors of CHF, and a Random Forest (RF) classifier assessed predictive importance of SDOH and demographic features. The Synthetic Minority Oversampling Technique (SMOTE) was applied to address class imbalance. Hierarchical clustering was performed to identify patterns of co-occurring social needs.

Results

Patients with CHF were significantly older and more frequently male and Black compared with patients without CHF (p < 0.001). Age was the strongest predictor of CHF, with adjusted odds increasing nearly tenfold among individuals aged ≥75 years compared with younger adults. Depression registry entry and financial insecurity increased odds, whereas being married or living with a partner was protective. All SDOH were significantly associated with CHF except tobacco use, although effect sizes were small. Random Forest achieved moderate performance (area under the receiver operating characteristic (ROC) curve (AUC) = 0.67, balanced accuracy = 62%), with age, transportation barriers, and physical activity ranking as top predictors. Clustering revealed distinct subgroups, including patients with co-occurring depression, alcohol use, stress, and another characterized by financial strain, food insecurity, and transportation limitations.

Conclusion

Demographic and social conditions contribute meaningfully to CHF burden, with age, socioeconomic stressors, and psychosocial vulnerability emerging as key factors. Despite small individual effect sizes, collective SDOH patterns demonstrated clinical relevance and predictive utility. Integrating SDOH variables into analytic models, supported by machine learning and oversampling techniques such as SMOTE, provides a scalable approach to improve CHF risk stratification and guide targeted intervention strategies.

## Introduction

Social factors, also referred to as social determinants of health (SDOH), help health systems and professionals classify and understand the diverse needs of their patients, determine suitable interventions to meet a variety of needs, and transform care to improve health outcomes [1]. It covers domains such as race/ethnicity, tobacco and alcohol use, educational background, financial stress, mental health, physical activity, social connections, domestic violence, and neighborhood income [2]. Incorporating SDOH into the clinical workflow enhances health outcomes while also reducing preventable expenses [3], particularly for patients with chronic health conditions, such as congestive heart failure (CHF).

CHF is the leading cause of hospitalization in older adults in the United States, and it accounts for one-fifth of deaths in the United States [4]. The upward trend of the disease has been tied to its chronic nature, which is heavily influenced by social factors that might be out of the healthcare providers’ view, but which contribute immensely to the management of the disease [5], hence the need for health institutions to collect and record SDOH needs. Leveraging SDOH data present in electronic health records (EHR) provides an opportunity to uncover social needs directly related to health. SDOH encompasses diverse social needs, which do not contribute equally to CHF outcomes.

Existing research on SDOH and heart failure is fragmented, analyzing factors in isolation rather than their combined impact [6-8]. To address this gap, this study utilizes healthcare analytics on a large, de-identified EHR dataset from a regional medical center to investigate the associations between documented SDOH factors and CHF status. By combining traditional statistical analysis with machine learning models, this explorative study asks, "To what extent are social determinants of health associated with congestive heart failure in inpatient populations, and how well can they contribute to risk discrimination?" Given the cross-sectional design, the objective is exploratory and hypothesis-generating, intended to inform future predictive and longitudinal modeling rather than infer causal relationships.

## Materials and methods

Study design and setting

This retrospective cohort study examined de-identified inpatient data from Our Lady of the Lake Regional Medical Center (OLOL), a large tertiary hospital in Baton Rouge, Louisiana. The dataset included all adult patients (≥18 years) hospitalized between January 1 and December 31, 2024, who were screened for social determinants of health (SDOH) using OLOL’s Epic electronic health record (EHR) system. Epic features an evidence-based SDOH questionnaire divided into four main domains [9], each with specific social risk indicators, as illustrated in Table 1. The study received approval from the Institutional Review Boards (IRBs) of both OLOL and Louisiana State University (LSU) (approval number: IRBAM-24-0865). 

**Table 1:** SDOH screening concerns captured by the Epic EHR platform EHR: electronic health record, SDOH: social determinants of health

Domain	Social determinants of health
Substance and sexual activity	Alcohol use
	Sexual activity
	Substance use
	Tobacco use
Socioeconomic	Demographics
	Financial resource strain
	Food insecurity
	Transportation needs
Lifestyle	Physical activity
	Stress
Relationships	Social connections
	Intimate partner violence

Data collection and de-identification

Patients with a CHF diagnosis were identified using the Chronic Disease Registry field in the EHR. The comparison group included all inpatients without an active CHF diagnosis but with chronic conditions that predispose to CHF, such as obesity, diabetes, or hypertension. De-identification followed HIPAA standards. Patient identifiers (names, medical record numbers, addresses, phone numbers, and full birthdates) were removed.

Birthdates were converted to age, which was categorized into 18-39 (young adults), 40-64 (middle-aged adults), and ≥65 (older adults), with the latter further divided into 65-74, 75-84, and ≥85 years. Each record was assigned a unique, randomly generated identifier for deduplication. The de-identified dataset contained 49,492 inpatient encounters, including 13,718 with CHF and 35,774 without CHF. Data were securely stored on the medical center’s restricted network and exported for analysis under institutional supervision. Data of interest included demographic information such as age, sex, and race, as well as SDOH needs captured in the screening questionnaires available on the EHR.

Data processing

To eliminate redundancy and ensure record-level integrity, duplicate entries for each patient were identified and removed. When multiple records existed for the same patient, the one with the most complete SDOH responses was kept. This resulted in 30,602 unique patient records, including 7,618 with CHF and 22,984 without CHF, which was the final dataset used for analysis in this study.

SDOH variables were selected based on clinical relevance and alignment with the health system’s standardized screening structure. Domains retained (financial strain, food insecurity, transportation, housing instability, stress, alcohol use, depression registry, social connection, physical activity, and abuse) were those most consistently documented in structured Epic fields to support generalizability. Selection, therefore, prioritized completeness and ecological validity rather than subjective researcher choice. Table 2 documents some of the questions and the needs addressed. Questions in asterisks were therefore selected to represent the presence or absence of the need in the analysis.

**Table 2:** SDOH questions documented in Epic EHR *Questions deemed by the researcher to capture best the SDOH need EHR: electronic health record, SDOH: social determinants of health

SDOH/social needs	Questions
Alcohol use	How often do you have a drink containing alcohol?
	*How many drinks containing alcohol do you have on a typical day when you are drinking?
	How often do you have six or more drinks on one occasion?
	Do you have a problem with alcohol or marijuana?
Substance use	Do you use medicine not prescribed to you, or any other types of drugs (such as cocaine, heroin, or meth)?
Tobacco use	Do you use tobacco or e-cigarettes?
Education	Do you have a high school degree?
	Do you ever need help reading hospital materials?
Demographics	Sex? Age? Race?
Financial resource strain	*How hard is it for you to pay for the very basics like food, housing, medical care, and heating?
	In the past 12 months, has the electric, gas, oil, or water company threatened to shut off services in your home?
Food insecurity	*Within the past 12 months, the food you bought just did not last, and you did not have the money to get more.
	Within the past 12 months, you worried that your food would run out before you got the money to buy more.
Housing insecurity	*In the last 12 months, was there a time when you were not able to pay the mortgage or rent on time?
	In the last 12 months, how many places have you lived?
	*In the last 12 months, was there a time when you did not have a steady place to sleep or slept in a shelter (including now)?
Transportation needs	*In the past 12 months, has a lack of transportation kept you from medical appointments or from getting medications?
	In the past 12 months, has a lack of transportation kept you from meetings and work, or from getting things needed for daily living?
Physical activity	*On average, how many days per week do you engage in moderate to strenuous exercise (like a brisk walk)?
	On average, how many minutes do you engage in exercise at this level?
Stress	*...Feel stressed, tense, restless, nervous, or anxious, or unable to sleep at night because your mind is troubled?
	Over the past two weeks, how often have you felt little interest or pleasure in doing things?
Social connections	*In a typical week, how many times do you talk on the phone with family, friends, or neighbors?
	*Are you married, widowed, divorced, separated, never married, or living with a partner?
	How often do you get together with friends or relatives?
	How often do you attend church or religious services?
	Do you belong to any clubs or organizations, such as church groups, unions, fraternal or athletic groups, or school groups?
	How often do you attend meetings of the clubs or organizations you belong to?
Intimate partner violence	*Within the last year, have you been humiliated or emotionally abused in other ways by your partner or ex-partner?
	*Within the last year, have you been raped or forced to have any kind of sexual activity by your partner or ex-partner?
	*Within the last year, have you been kicked, hit, slapped, or otherwise physically hurt by your partner or ex-partner?
	Within the last year, have you been afraid of your partner or ex-partner?

All SDOH variables were binary-coded as documented (Yes) versus not documented (No) for ease of analysis. Responses to the social determinants of health (SDOH) questions were recoded to facilitate analysis. For the item “How hard?”, responses of “hard”, “somewhat hard”, “very hard”, or “not very hard” were coded as “Yes,” while “not hard at all” was coded as “No”. Similarly, for “How true?”, responses of “often true” or “sometimes true” were coded as “Yes”, and “never true” was coded as “No”. For “How often do you have a need?”, responses indicating “only a little”, “rather much”, or “very much” were classified as “Yes”, while “not at all” was coded as “No”. Alcohol use was coded as “Yes” if the patient reported drinking “1 to 2 times”, “3 to 4 times”, “5 or 7 to 9 times”, or “10 or more times” per week, and “No” if the patient reported “not drinking” or “drinking 0 times per week”. Exercise frequency was coded as “Yes” for patients who exercised “more than three times a week”, “once a week”, “three times a week”, or “twice a week”. In all variables, responses where the patient declined, refused to answer, or left the field blank were coded as “Unknown”.

Statistical analysis

Descriptive statistics summarized demographic and SDOH distributions across CHF and non-CHF groups. Bivariate associations were tested using the chi-square (χ²) test of independence. Chi-square (χ²) is a statistical tool used to examine the independence between two categorical variables or to determine the goodness of fit [10]. For each significant χ² result, Cramér’s V was calculated to measure effect size, interpreted per Cohen’s criteria as small (0.10), moderate (0.30), or large (0.50) [11]. Analyses were conducted using IBM SPSS Statistics version 29 (IBM Corp., Armonk, NY).

To adjust for confounding, a multivariable logistic regression model was fitted with CHF status as the outcome and demographic variables (age, sex, and race) and SDOH factors as predictors. Because the non-CHF cohort already included patients with CHF-related comorbidities, these were not entered as covariates. Adjusted odds ratios (aORs) and 95% confidence intervals (CIs) were reported. Model adequacy was evaluated using the Hosmer-Lemeshow test, variance inflation factors (VIF), and McFadden’s pseudo-R².

Machine learning analysis

To investigate the predictive relationship between SDOH and CHF status, a supervised machine learning model was developed using the Random Forest (RF) algorithm. The Random Forest (RF) analysis was performed in R (version 4.4.0) using the randomForest package. The model was trained on a dataset comprising demographic variables (age group, sex, and race), as well as the SDOH indicators (alcohol use, food insecurity, housing instability, financial instability, abuse history, stress, tobacco use, and depression). The binary outcome variable was CHF status (1 = CHF, 0 = non-CHF). The choice of Random Forest is justified by its strength in handling large datasets with multiple predictor variables and its ability to model complex interactions and nonlinear relationships [12]. Missing categorical values were coded as “Unknown” and retained. Data were split into 70% training and 30% test sets.

The Random Forest model tested combinations of 100-500 trees and maximum depths ranging from 10 to 1,000. Each configuration was evaluated using five-fold cross-validation on the training set [13]. The model that achieved the highest mean cross-validated area under the receiver operating characteristic (ROC) curve (AUC) was selected as the final configuration. To address class imbalance between patients with CHF and without CHF, the Synthetic Minority Oversampling Technique (SMOTE) was applied to the training dataset after tuning. SMOTE synthesizes new minority class samples by interpolating between nearest neighbors, thereby enhancing model learning without duplicating observations [14]. Feature importance was assessed using both permutation importance and mean decrease in impurity (MDI). Model evaluation incorporated accuracy, precision, recall, F1 score, AUC-ROC, Cohen’s Kappa, and Brier score for calibration.

Visualization

In addition to the above analysis, a heatmap with dendrograms was generated to visualize the relationship between patients with CHF and the SDOH needs. The core of the heatmap is a matrix that represents the individual patient with CHF, with columns labeled with SDOH factors/ needs, and each cell within the matrix color-coded to indicate the presence (red) or absence (green) or unknown (gray) of a particular SDOH need for a given patient. The dendrograms on the left vertical axis of the heatmap reveal the similarities among the rows (patients) and columns (SDOH needs) through data clustering.

**Figure 1:**
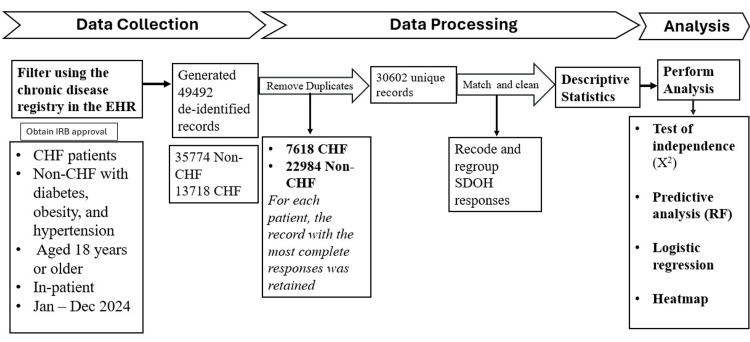
Data processing workflow/methodology of the study CHF: congestive heart failure, EHR: electronic health record, IRB: institutional review board, SDOH: social determinants of health, RF: Random Forest, X2: chi-square

Figure 1 outlines the detailed methodology for this study.

## Results

Demographic characteristics of the study population

The analytic dataset included 30,534 unique inpatient records, comprising 7,618 patients with CHF and 22,916 without CHF. Across SDOH variables, nearly all domains showed significant associations with CHF (p < 0.001) as documented in Table 3.

**Table 3:** Demographic characteristics of patients with and without CHF CHF: congestive heart failure

Variable	CHF (n = 7,618)	Non-CHF (n = 7,618)	Total (n = 15,236)	Chi-square (χ2)	p-value
Age group				1905	<0.001
18-39	230 (3.0%)	3,560 (15.5%)	3,790 (12.4%)		
40-64	2,220 (29.1%)	9,299 (40.5%)	11,519 (37.6%)		
65-74	2,135 (28.0%)	5,441 (23.7%)	7,576 (24.8%)		
75-84	2,036 (26.7%)	3,549 (15.4%)	5,585 (18.3%)		
85+	997 (13.1%)	1,132 (4.9 %)	2,129 (7.0%)		
Sex				28.4	<0.001
Male	4,085 (53.6%)	11,528 (50.2%)	15,613 (51%)		
Female	3,533 (46.4%)	11,453 (49.8%)	14,986 (49%)		
Race				80.84	<0.001
White	4,106 (53.9%)	13,028 (56.7%)	17,134 (56%)		
Black	3,269 (42.9%)	8,802 (38.3%)	12,071 (39.4%)		
Other	243 (3.2%)	1,154 (5%)	1,397 (4.6%)		

Patients with CHF were significantly older than those without CHF (χ² = 1905, p < 0.001). Among patients with CHF, 67.8% were aged 65 years or older, compared to only 43.6% in the non-CHF group. The proportion of patients with CHF increased noticeably with age, underscoring the strong age-related nature of CHF, with prevalence increasing progressively across age brackets.

Sex differences across groups were modest but statistically significant (χ² = 28.4, p <0.01). Males comprised a greater proportion of the CHF group (53.6%) compared to the non-CHF group (50.2%). Conversely, females were slightly overrepresented in the non-CHF group (49.8%).

Racial disparities were also evident (χ² = 80.84, p < 0.001). Black patients constituted a larger share of the CHF group (42.9%) compared to the non-CHF group (37.9%), while White patients made up a smaller proportion of patients with CHF (53.9%) than those without CHF (57.2%). Individuals who were not white/Caucasians or black/African Americans were grouped as “Other races”. This group represented a minor portion of both groups but was more prevalent in the non-CHF group (4.9%) than in the CHF group (3.2%).

Test of independence using chi-square

The test of independence in Table 4 reveals that alcohol use, housing instability, and marital status have a more significant association (p < 0.001) with CHF status compared to the other needs, with effect sizes (Cramer's V) of 0.142, 0.066, and 0.046, respectively. Abuse-related factors, social connection, and transportation have a lesser significance association (p < 0.001) with CHF status, with weak effect sizes (Cramer's V) of 0.044, 0.041, and 0.038, respectively. Financial insecurity, depression, physical activity, and food insecurity had the least significant association (p < 0.001) with CHF status, with effect sizes (Cramer's V) of 0.023, 0.025, 0.031, and 0.033, respectively. Tobacco use was the only SDOH variable that had no association with CHF status (χ² = 1.15, p = 0.28, Cramér’s V = 0.006).

**Table 4:** Test of independence/effect size of SDOH to CHF status CHF: congestive heart failure, SDOH: social determinants of health

SDOH	Chi-square (χ²)	p-value	Effect size	Unknowns
Depression	19.28	<0.001	0.025	0
Tobacco use	1.151	0.283	0.006	0
Housing stability	134.28	<0.001	0.066	61.40%
Financial constraint	15.54	<0.001	0.023	66.40%
Sexual abuse	51.7	<0.001	0.041	32.3
Physical abuse	58.7	<0.001	0.044	32.20%
Transportation barrier	43.57	<0.001	0.038	27.90%
Food insecurities	34.21	<0.001	0.033	28.20%
Stress	34.14	<0.001	0.033	74.40%
Social connections	51.71	<0.001	0.041	28.80%
Marital status	63.96	<0.001	0.046	71.40%
Physical active	30.163	<0.001	0.031	84.00%
Alcohol use	618.402	<0.001	0.142	23.50%

The chi-square tests of independence yielded predominantly statistically significant results, indicating significant associations between CHF status and all SDOH except tobacco use. However, the practical or clinical relevance of these associations may be limited. According to Cohen’s thresholds for interpreting Cramér’s V, which classifies values less than 0.30 as small effects, all the examined variables demonstrated small to negligible effect sizes, which might be due to the level of completeness of the SDOH in EHR.

Multivariable logistic regression

The multivariable logistic regression, adjusting for demographics and SDOH, identified several significant predictors of CHF (Table 5). Compared with younger adults, the odds of CHF increased progressively with age: 40-64 years (aOR = 3.97, 95% CI = 2.25-7.58), 65-74 years (aOR = 4.35, 95% CI = 2.42-8.45), 75-84 years (aOR = 9.44, 95% CI = 5.22-18.4), and ≥85 years (aOR = 9.65, 95% CI = 4.93-20.1). Male sex was modestly associated with higher odds (aOR = 1.27, p = 0.049). Depression registry entry (aOR = 1.44, p = 0.002) and financial insecurity (aOR = 1.15, p = 0.03) were also significant predictors. Conversely, being married or living with a partner was protective (aOR = 0.63, p < 0.001), as was alcohol use (aOR = 0.71, p = 0.008). White patients had lower adjusted odds of CHF compared to non-White patients (aOR = 0.71, p = 0.005).

**Table 5:** Adjusted logistic regression predictors of CHF status 95% CI: 95% confidence interval (highest-lowest), aOR: adjusted odds ratio, CHF: congestive heart failure

Variable	aOR	95% CI	p-value
Age (40-64)	3.97	2.25-7.58	<0.001***
Age (65-74)	4.35	2.42-8.45	<0.001***
Age (75-84)	9.44	5.22-18.4	<0.001***
Age (≥85)	9.65	4.93-20.1	<0.001***
Male (sex)	1.27	1.00-1.62	0.049*
Race (White)	0.71	0.56-0.90	0.005**
Race (Other)	0.55	0.28-1.02	0.070
Tobacco use	1.05	0.81-1.37	0.709
Depression	1.44	1.14-1.83	0.002**
Housing insecurity	1.31	0.86-1.99	0.197
Financial insecurity	1.15	0.87-1.52	0.326
Sexually abused	≈ 0.00	-	0.976
Physically abused	0.71	0.07-7.47	0.768
Transportation barrier	1.06	0.68-1.67	0.786
Food insecurity	1.01	0.64-1.60	0.958
Stressed	1.04	0.81-1.34	0.791
Social connection	0.93	0.50-1.71	0.824
Married/living with partner	0.63	0.49-0.80	<0.001***
Physically active	0.8	0.63-1.02	0.072
Alcohol use	0.68	0.52-0.89	0.008**

Predictive modeling (Random Forest)

The Random Forest model achieved an AUC of 0.67, indicating moderate discrimination. On the unbalanced test set, the model produced an accuracy of 66.3%, sensitivity of 53.5%, specificity of 70.5%, balanced accuracy of 62.0%, and Cohen’s κ = 0.21, indicating fair agreement beyond chance. The F1 score was 0.76. The calibration curve demonstrated moderate reliability, with a Brier score of 0.22, reflecting acceptable calibration across predicted risk bins.

The confusion matrix in Table 6 reveals that the model correctly classified 67% of CHF cases (sensitivity) and 57.6% of non-CHF cases (specificity). The positive predictive value (i.e., precision for CHF prediction) was 61.2%, while the negative predictive value (non-CHF prediction) was 63.6%. The balanced accuracy, accounting for both sensitivity and specificity, was 62% (Table 7).

**Table 6:** Confusion matrix on the test variables (N = 9,180)

Prediction	Non-CHF	CHF
Non-CHF	4,863	1,063
CHF	2,032	1,222

**Table 7:** Predictive performance metrics for the random forest model CI: confidence interval, NPV: negative predictive value, PPV: positive predictive value

Metric	Value
Accuracy	0.6629
95% CI	(0.6531, 0.6725)
No information rate	0.7511
p-value [Acc > NIR]	1
Kappa	0.2103
McNemar’s test p-value	7.251e−07
Sensitivity	0.5348
Specificity	0.7053
PPV	0.3755
NPV	0.8206
Prevalence	0.2489
Detection rate	0.1331
Detection prevalence	0.3545
Balanced accuracy	0.620

**Figure 2:**
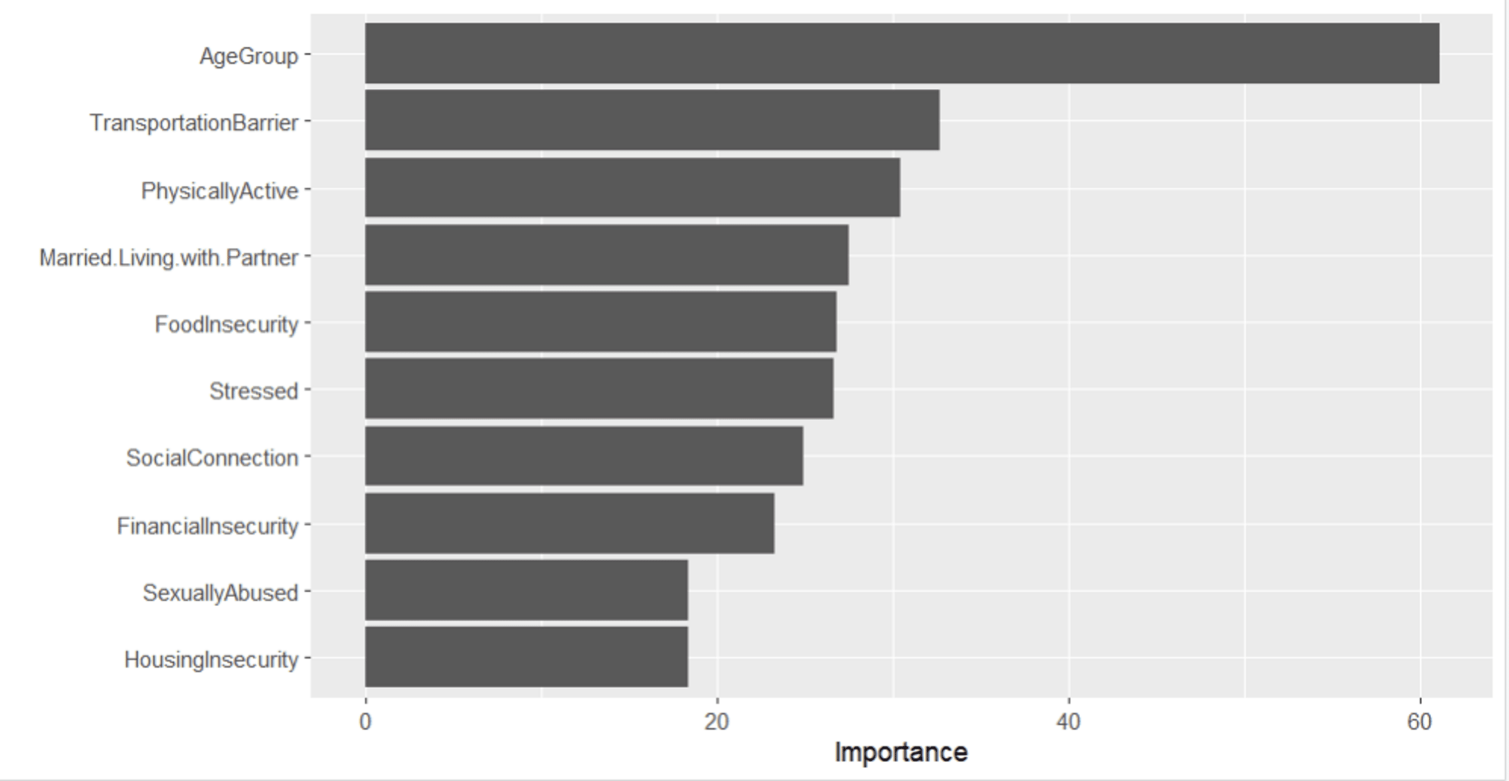
Relative importance of SDOH variables in predicting CHF using the Random Forest model CHF: congestive heart failure, SDOH: social determinants of health

Permutation-based feature importance identified age group, transportation barrier, and physical activity as the strongest predictors, followed by marital status, food insecurity, stress, social connection, and financial insecurity (Figure 2). The three least contributors were financial insecurity, sexual abuse, and housing insecurity.

Heatmap and clustering of SDOH needs among patients with CHF

**Figure 3:**
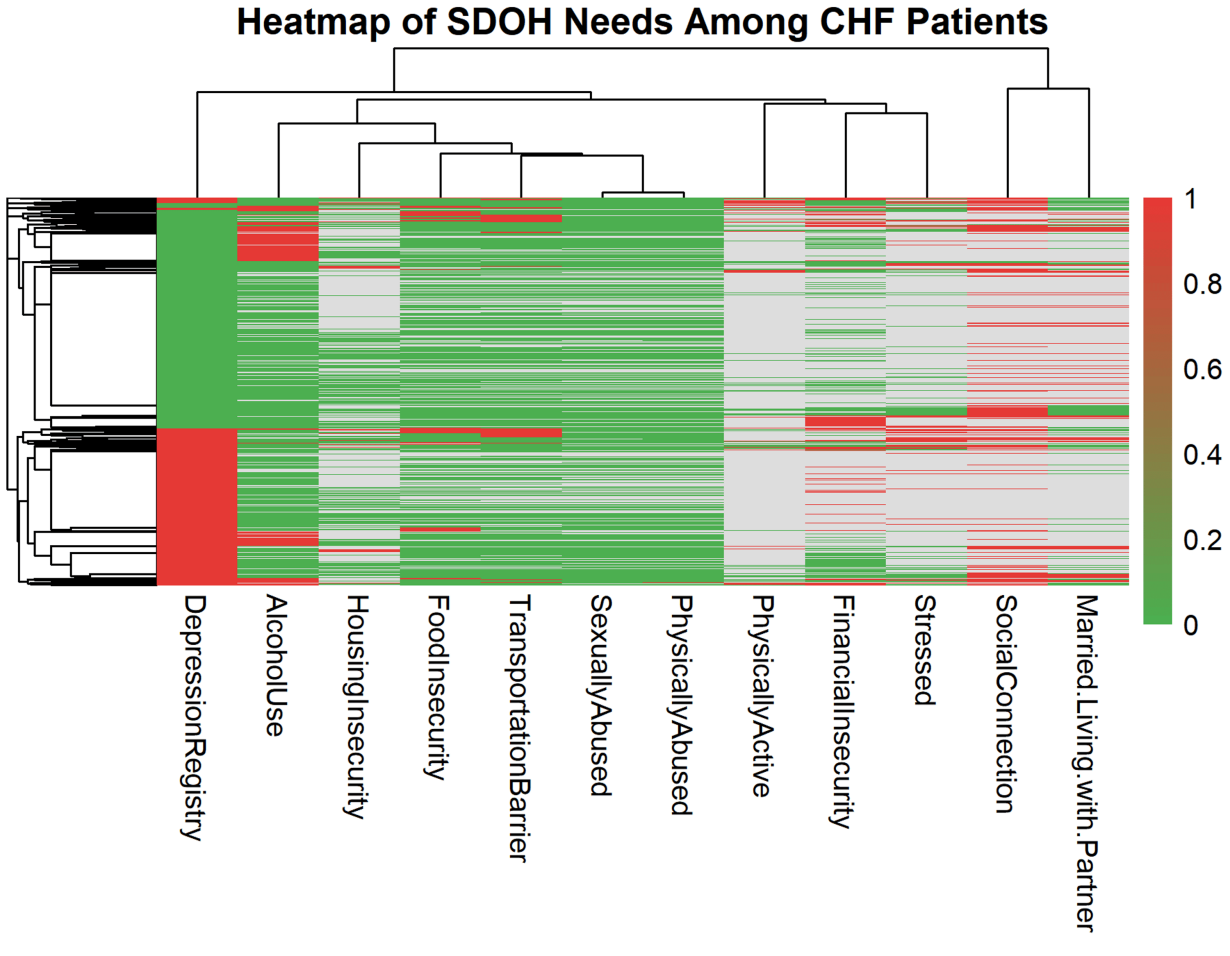
Heatmap and clustering of SDOH needs among patients with CHF Green: absence of SDOH, red: presence of SDOH, gray: unknown SDOH CHF: congestive heart failure, SDOH: social determinants of health

Hierarchical clustering was applied to both dimensions, revealing patterns of co-occurrence between specific SDOH factors and patient subgroups (Figure 3). Distinct clustering patterns emerged across the 12 SDOH variables. Depression registry entries and alcohol use were the most prevalent needs, forming a dense red cluster on the left of the heatmap. Financial and housing insecurity frequently appeared together, suggesting that economic hardship may underlie multiple social stressors among patients with CHF. Similarly, food insecurity, transportation barriers, and stress clustered in proximity, indicating a potential behavioral environmental linkage affecting disease management and follow-up adherence. In contrast, social connection and marital status exhibited higher proportions of green cells, signifying comparatively lower documentation of social isolation in this cohort.

The row dendrogram demonstrated several patient subgroups with overlapping psychosocial profiles. One major cluster contained patients with concurrent depression, alcohol use, and stress, while another grouped those with financial strain, food insecurity, and transportation challenges. These subgroupings imply differentiated social burden patterns within the CHF population. 

## Discussion

This study analyzed 30,534 inpatients to explore the association between SDOH and CHF using real-world EHR data from a large regional medical center. Both statistical and machine learning techniques were employed to identify patterns that could inform targeted interventions for the management of CHF. The results expand current understanding of the social context of CHF by quantifying the relative contribution of multiple SDOH domains to CHF risk.

CHF, the final stage of all cardiovascular diseases, does not imply that the heart’s functionality has completely failed. It is a condition that results in the heart being unable to pump enough blood to meet the body’s blood requirements [15]. There is no known cure for CHF; however, its treatment depends on early detection and effective management [16]. Early detection of symptoms enables interventions to be implemented as soon as possible, thereby successfully containing their progression or advancement [17].

The findings reinforce the well-established age-related nature of CHF; almost two-thirds of patients with CHF were aged 65 years or older, compared with less than half of patients without CHF. The odds of CHF increased markedly with age in the logistic regression, demonstrating a nearly tenfold increase among patients 75-84 years and those aged 85 years or older relative to younger patients. This age gradient aligns with existing evidence indicating that aging is one of the strongest non-modifiable risk factors for CHF [18].

Sex and race also contributed meaningfully to disease distribution. Male sex was associated with a modest elevation in odds of CHF, consistent with prior observations that men often experience earlier onset of clinical symptoms. Racial patterns suggested heightened disease burden among Black patients, which may reflect structural disparities in cardiovascular risk exposure, healthcare access, and accumulated disadvantage. Conversely, White patients had significantly lower adjusted odds of CHF. These findings underscore the ongoing need to assess racial disparities in disease management and access to preventive resources, as well as risk stratification increment, especially when combined with comorbidity profiles or longitudinal clinical features in future work.

Nearly all SDOH domains were significantly associated with CHF status based on chi-square tests, although effect sizes were small. Alcohol use, housing instability, and marital status exhibited the strongest associations. Notably, alcohol use and depression registry entries emerged as key predictors in the regression model. Marriage or partnership was protective, aligning with literature describing the buffering effect of social support on disease management and recovery [19]. Interestingly, alcohol use appeared protective in adjusted models, a pattern that warrants cautious interpretation. Possible explanations include documentation bias, selective disclosure, or confounding by social engagement. Tobacco use was the only variable with no association with CHF, which may reflect limitations in EHR capture rather than a true absence of effect.

These statistically significant but clinically weak associations might imply that most SDOH impacts on CHF may be indirect or develop over long periods. The use of structured response formats might limit the ability to fully capture the complexity and persistence of social challenges. Incorporating unstructured data, such as narrative responses, could offer deeper insights into how social factors influence CHF risk. Small effect sizes are typical in large studies, where statistical significance does not always mean clinical importance [20]. This may be due to the sensitivity of SDOH questions, data collection methods, and the complex causes of CHF, where biological, behavioral, and social risks interact.

Machine learning analysis using Random Forest demonstrated moderate discrimination, with an AUC of 0.67 and balanced accuracy of 62%, indicating that SDOH variables alone provide limited predictive strength for CHF classification. Rather than suggesting clinical deployment, these results highlight the exploratory value of integrating social factors into analytic models as a complement to clinical data rather than a replacement for it. Age group, transportation barriers, and physical activity emerged as the most influential features in the model, suggesting potential relevance for future risk stratification frameworks, although these relationships should be interpreted as associations rather than determinants.

Hierarchical clustering revealed descriptive patterns of co-occurring social needs, including depression with alcohol use and stress, and financial strain with food or housing insecurity. While not directly prescriptive, these subgroup patterns help illustrate how social vulnerabilities tend to cluster within CHF populations and may guide future hypothesis-driven work on targeted support strategies.

Overall, the findings demonstrate that demographic and social conditions jointly contribute to CHF risk and reveal distinct psychosocial clusters that could inform care coordination strategies. While the statistical associations were small in magnitude, even modest effects may have clinical value when applied across large populations, particularly in triage and prevention workflows.

Limitations and future research

This study is not without limitations. First, reliance on routinely collected EHR data may introduce documentation bias and missingness, particularly within SDOH fields that are captured inconsistently across clinical encounters. Small effect sizes for several variables likely reflect incomplete reporting rather than the absence of influence. Second, this analysis was observational, and the results cannot infer causal relationships between social conditions and CHF. Third, model performance, although moderate, may improve with additional clinical variables, longitudinal trajectories, or unstructured clinical note integration. Fourth, the study was conducted within a single regional health system, which may limit generalizability beyond similar demographic and practice contexts.

Future research should expand this work by using multi-institutional datasets to improve generalizability. In addition, more advanced machine learning approaches such as XGBoost, gradient boosting machines, or neural network-based models may help evaluate whether predictive performance can be improved beyond Random Forest. Enhanced SDOH capture through structured screening, improved completeness, or natural language processing of unstructured notes may also increase model sensitivity and deepen understanding of how social complexity contributes to heart failure outcomes.

## Conclusions

This study demonstrates that while SDOH are statistically associated with CHF, effect sizes were small and predictive power was moderate, indicating that social factors provide incremental insight rather than strong standalone indicators of disease burden. These findings should be interpreted as exploratory, generating hypotheses for how social needs cluster and how they may contribute to future risk stratification frameworks when combined with clinical variables. Rather than drawing clinical or causal conclusions, the dual-method approach highlights the value of integrating EHR-derived SDOH in analytic pipelines. It lays the groundwork for future model refinement, multi-site validation, and assessment of temporal risk patterns. Continued work is needed to evaluate whether incorporating additional clinical features and longitudinal EHR trajectories can enhance predictive accuracy and support actionable intervention strategies.
